# Robust Cooperative Moving Path Following Control for Marine Robotic Vehicles

**DOI:** 10.3389/frobt.2019.00121

**Published:** 2019-11-21

**Authors:** Matheus F. Reis, R. Praveen Jain, A. Pedro Aguiar, Joao Borges de Sousa

**Affiliations:** Department of Electrical and Computer Engineering, Faculty of Engineering, University of Porto, Porto, Portugal

**Keywords:** marine robotics, underactuated robotics, path following, robust control, cooperative control

## Abstract

This paper presents results on recent developments pertaining to the coordinated motion control of a fleet of marine robotic vehicles. Specifically, we address the Cooperative Moving Path Following (CMPF) motion control problem, that consists of steering the robotic vehicles along a priori specified geometric paths that jointly move according to a target frame, while achieving a pre-defined coordination objective. To this end, each vehicle will need to communicate with their neighbors in order to cooperatively solve the CMPF task. Two distinct robust Moving Path Following motion control strategies for achieving robustness on the moving path following tasks are proposed. Experimental results demonstrating the application of CMPF to marine vehicles in the context of source localization and tracking of underwater targets are presented backed with stability and convergence guarantees.

## 1. Introduction

The motion control problem for underactuated robotic vehicles is a relatively mature area of research, with important works addressing trajectory tracking and path following schemes. In the path following (PF) problem, the vehicle is tasked to follow a *fixed* geometric path without the need of satisfying explicit time constraints, in contrast to trajectory tracking. A series of results addressing the PF motion control problem were published, starting with the pioneering work in Samson (1992); Micaelli and Samson (1993), and Aguiar and Pascoal (2007) for the case of wheeled mobile robots, Encarnação et al. (2000); Belleter et al. (2016) and references therein for marine vehicles and Cichella et al. (2011); Xargay et al. (2013) for UAVs.

A generalization of the path following problem is termed the Moving Path Following (MPF) motion control problem, which consists of steering the robotic vehicle along an a priori specified geometric path expressed with respect to a *moving* target frame. This problem finds applications in source seeking, convoy protection, target tracking, surveillance and monitoring and also autonomous landing. For example, in target tracking applications in the maritime environment, it is desirable for the vehicles to perform different types of maneuvers. These maneuvers can be framed as specific paths to be followed around the tracked target, and often allow the vehicle to have the necessary flexibility to operate in a highly complex environment, which is constantly inducing disturbances into its body due to the presence of maritime currents, waves and hydrodynamic effects. Further, it is observed that the MPF problem retains the advantages of the classical path following schemes (Aguiar et al., 2005) such as faster convergence of the robot to the moving path, while allowing the target reference frame to move freely. The works in Oliveira and Encarnação (2013) and Oliveira et al. (2016) introduced the MPF control problem for tracking of ground targets using Unmanned Aerial Vehicles (UAVs) and later on, Oliveira et al. (2017) extended the solution to the 3D case. The proposed approach was suitable for robotic vehicles requiring a minimum positive forward speed, such as certain types of AUVs. In Jain et al. (2018b), a Lyapunov-based MPF control approach was presented for robotic vehicles without this restriction. Other control methods such as vector field method (Kapitanyuk et al., 2017) and nonlinear model predictive control (Jain et al., 2018c) have been proposed to solve the MPF problem. In contrast to the contributions of this paper, the salient features of the above reviewed literature are that they do not consider the external disturbances that depend on the operational environment, such as maritime currents, wind or rough terrain, that can affect the performance of the MPF controller. Further, they assume that the velocity of the target frame is known.

In path following literature, the problem of robustness has been addressed for example, in Dagci et al. (2003), where a cascade sliding mode controller for both kinematics and dynamics of a robotic vehicle was designed. In Aguiar and Pascoal (2002), a disturbance observer for constant unknown ocean currents was designed to solve the problem of dynamic positioning and way-point tracking of an underactuated AUV. Later on, the problem of robustness against parametric uncertainty in trajectory tracking and path following was also addressed in Aguiar and Hespanha (2007). More recently, in Zhang et al. (2014), a sliding mode technique combined with a predictive control strategy was developed to compensate for the impact of the hydrodynamic damping coupling on a 3D path following task for an Autonomous Underwater Vehicle (AUV). In Wang et al. (2016), a *H*_∞_ robust controller for ground vehicles is proposed to achieve path following in the presence of disturbances caused by delays and data packet dropouts. All of the above schemes consider robustness for the path following problem. From the best of the authors knowledge, the only work concerning the problem of robustness in MPF literature is Reis et al. (2019), where sliding mode based controllers and a disturbance observer were designed to compensate external disturbances acting on the robotic vehicle.

A further extension of the MPF framework for multi-robot applications and formation control is the Cooperative Moving Path Following (CMPF) control problem, which consists in steering *N* vehicles along *N* paths defined with respect to a moving target while achieving some coordination objective. A special case of CMPF control, where the paths are fixed with respect to a given reference frame is the framework of Cooperative Path Following (CPF). As a recent example, the robustness problem in CPF literature was addressed in Gu et al. (2019), where two cooperative path following controllers using an Extended State Observer to estimate and compensate external disturbances in the kinetic level were proposed and validated experimentally using Autonomous Surface Vehicles (ASVs). In (Jain et al., 2018a), an event-based controller was explicitly designed to reduce the frequency of communication between the robotic vehicles. The control strategy effectively decomposes the control structure into two distinct layers. The first is responsible for the motion control of each individual vehicle, termed the PF controller. The second, termed the cooperative controller, is responsible for achieving coordination between the robots by using a consensus law. However, (Jain et al., 2018a) does not consider uncertainties and disturbances acting on the robotic vehicles. By decoupling the motion control layer from the cooperative control layer, one could use robust MPF controllers in the first layer to deal with the presence of certain types of disturbances acting on the vehicles, without affecting the formation control.

This paper extends the results obtained for the MPF controllers proposed by Reis et al. (2019) to the Cooperative MPF framework. Two MPF control strategies are proposed for the motion control layer, both using a known target pose and estimates of the target velocities. The first strategy employs a First Order Sliding Mode (FOSM) term to achieve robustness against bounded disturbances. The second strategy seeks to directly compensate the disturbance by computing an estimate of the disturbance using a disturbance observer. The cooperative layer consists of the consensus law proposed by Aguiar (2017). The stability of the proposed control laws is analyzed and it is shown that the origin of the path error is stable and converges to a small neighborhood around zero, even in the presence of bounded estimation errors on the target velocities and environmental disturbances. The design and theoretical results for the two variants of the proposed robust controllers are experimentally validated in a CMPF scenario using Autonomous Underwater Vehicles.

## 2. Problem Formulation

### 2.1. Kinematic Model for an Underactuated Vehicle

Consider an inertial frame of reference {*I*} and *N* robotic vehicles, each with its body frame {*B*_*i*_} attached to its center of mass. Define the set of *N* robotic vehicles as I={1,2,…,N}. The kinematic model of the *i*-th vehicle moving in ℝ^*n*^ with *n* = 2, 3 can be expressed by

(1) $$\begin{array}{l} {\dot{p}}_{i}\left(t\right)=R_{i}\left(t\right)v_{i}+d_{v,i}\\ {\dot{R}}_{i}\left(t\right)=R_{i}\left(t\right)S\left(\omega_{i}+d_{\omega ,i}\right) \end{array}$$

where $p_{i}\in {\mathbb{R}}^{n}$ denotes the position of the *i*-th robot with respect to the frame {*I*}, $R_{i}\in SO\left(n\right)$ denotes the rotation matrix from the frame {*B*_*i*_} to an inertial frame {*I*}, $v_{i}\in {\mathbb{R}}^{n}$ and $\omega_{i}\in {\mathbb{R}}^{n\left(n-1\right)/2}$ are the linear and angular velocities of the *i*-th vehicle with respect to its own body frame, *S*(**ω**_*i*_) ∈ 𝔰𝔬(*n*) is the skew-symmetric matrix associated to the angular velocity **ω**_*i*_.

Finally, $d_{v,i}\in {\mathbb{R}}^{n}$ and $d_{\omega ,i}\in {\mathbb{R}}^{n\left(n-1\right)/2}$ are kinematic disturbances acting on each robot. Many different factors can be the source of these disturbances, depending on the type of vehicle and the operational environment. Marine vehicles such as AUVs are affected by unknown sea conditions that can induce unwanted external velocities due to maritime currents, waves and wind. In the case of aerial vehicles, wind and internal dynamics can induce some unwanted disturbances in the kinematic model. In this work, we consider the problem of controlling an underactuated vehicle at the kinematic level, with the control signal defined as

(2) $$\begin{array}{l} u_{i}=\left[\begin{array}{c} v_{f,i}\\ \omega_{i} \end{array}\right] \end{array}$$

where the body linear velocity ***v***_*i*_ is defined as $v_{i}={\left[v_{f,i}\;0\right]}^{T}$ (*n* = 2) or $v_{i}={\left[v_{f,i}\;0\;0\right]}^{T}$ (*n* = 3). This is the case for vehicles where only the longitudinal velocity *v*_*f, i*_ ∈ ℝ and the body angular velocity $\omega_{i}\in {\mathbb{R}}^{n\left(n-1\right)/2}$ can be controlled, such as some types of AUVs. We assume that the vehicle has an inner-loop autopilot controller that is responsible to track the linear and angular velocity commands generated by the controller based on the kinematic model of the robotic vehicle. Imperfect tracking by the inner-loop autopilot controller can further contribute to the velocity disturbances acting on the vehicle, that can be lumped into the terms ***d***_*v, i*_ and ***d***_ω, *i*_.

### 2.2. Cooperative Moving Path Following Problem

In the CMPF control problem, the vehicles must follow a priori specified paths expressed with respect to a moving target whose position can be accurately estimated, while also maintaining some coordination objective. Define the target frame {*T*} with its origin attached to the target center of mass. Then, the cooperative MPF problem can be divided in the following two sub-problems.

#### 2.2.1. Moving Path Following Problem

**Figure 1:**
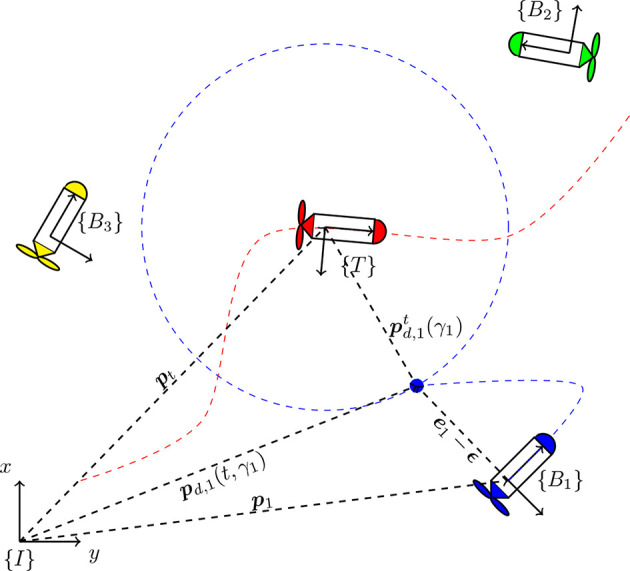
Coordinate frames and vector notation for *N* = 3 vehicles.

Let $p_{t}\left(t\right)\in {\mathbb{R}}^{n}$ denote the position of the target with respect to the frame {*I*}, and $p_{d,i}^{t}\left(\gamma_{i}\right)\in {\mathbb{R}}^{n}$ be the desired path for vehicle *i*, specified with respect to the frame {*T*} and parameterized by the path variable γ_*i*_ ∈ ℝ. As illustrated by Figure 1, for a given γ_*i*_ and time *t*, ***p***_*d, i*_(γ_*i*_, *t*) and ${\overset{∙}{p}}_{d,i}\left(\gamma_{i},t\right)$ denote the position and velocity of the virtual reference point that must be followed by the *i*-th vehicle, with respect to the inertial frame {*I*}:

(3) $$\begin{array}{l} p_{d,i}\left(\gamma_{i},t\right)=p_{t}+R_{t}p_{d,i}^{t} \end{array}$$

(4) $$\begin{array}{l} {\overset{∙}{p}}_{d,i}\left(\gamma_{i},t\right)=v_{t}+R_{t}\left(\nabla p_{d,i}^{t}{\overset{∙}{\gamma }}_{i}+S\left(\omega_{t}\right)p_{d,i}^{t}\right) \end{array}$$

where $R_{t}\left(t\right)\in SO\left(n\right)$ is the rotation matrix of frame {*T*} with respect to {*I*}, $v_{t}\left(t\right)\in {\mathbb{R}}^{n}$, $\omega_{t}\left(t\right)\in {\mathbb{R}}^{n\left(n-1\right)/2}$ are the linear and angular target velocities and ∇ ≡ ∂/∂γ_*i*_ is the derivative with respect to γ_*i*_.

Assumption 2.1. *The geometric path* $p_{d,i}^{t}\left(\gamma_{i}\right)$ *is a differentiable function.*

Note that Assumption 2.1 is already needed in order to compute (4) from (3). Suppose we wish to control the position of the nose of the *i*-th vehicle, or more generically, a point ${\bar{p}}_{i}=p_{i}+R_{i}\epsilon$ placed at a constant position $\epsilon ={\left[\epsilon_{1\;}\epsilon_{2\;}\right]}^{T}$ (*n* = 2) or $\epsilon ={\left[\epsilon_{1\;}\epsilon_{2\;}\epsilon_{3\;}\right]}^{T}$ (*n* = 3) from the origin of {*B*_*i*_}. Then, define the MPF error associated to the *i*-th vehicle as the vector

(5) $$\begin{array}{l} e_{i}=R_{i}^{T}\left({\bar{p}}_{i}-p_{d,i}\right),\;i\in I \end{array}$$

The objective of the MPF control problem is to design a control law ***u***_*i*_ such that the origin ***e***_*i*_ ≡ 0 is stable and ***e***_*i*_ → 0 as *t* → ∞, ∀i∈I. That is, it is desired to steer the vehicles toward their moving geometric paths, such that ${\bar{p}}_{i}$ stabilizes around ***p***_*d, i*_(γ_*i*_, *t*), ∀i ∈ I.

In order to control the progression of the virtual points ***p***_*d, i*_(*t*, γ_*i*_) along the moving paths, the dynamics of the path variable ${\overset{∙}{\gamma }}_{i}$ should be explicitly controlled. This can be achieved by imposing the dynamics for γ_*i*_ as

(6) $$\begin{array}{l} {\overset{∙}{\gamma }}_{i}=v_{d}+\vartheta_{i},\;i\in I \end{array}$$

where the scalar *v*_*d*_ is the desired nominal speed of the path variable and ϑ_*i*_ is a bounded control signal, designed to achieve CMPF objectives such as: (i) consensus over the path variables of the robotic vehicles to achieve a desired formation along the moving path and (ii) faster convergence to the moving path. To move along the geometric paths with the desired velocity, the vehicles must satisfy the desired speed assignments $|{\overset{∙}{\gamma }}_{i}-v_{d}|\to 0$ as *t* → ∞, ∀i∈I.

#### 2.2.2. Cooperative Motion Control Problem

Assume that the *i*-th vehicle communicates with a fixed set $N_{i}\subset \mathbb{N}$ of neighbor vehicles. Given the path variables γ_*i*_ (i∈I) for the *N* vehicles and a given undirected, fixed communication topology among them, the objective of the cooperative motion control problem is to design a decentralized control law *v*_*r, i*_(*t*) such that the positions of the virtual points are synchronized, that is, |γ_*i*_ − γ_*j*_| converges to zero ∀i,j∈I as *t* → ∞. To simultaneously achieve the speed assignment, coordination objective and also two other secondary objectives, function ϑ_*i*_ in (6) is decomposed as

(7) $$\begin{array}{l} \vartheta_{i}=v_{r,i}\left(t\right)+g_{e,i}\left(t\right)+g_{\omega ,i}\left(t\right) \end{array}$$

where *v*_*r, i*_(*t*) is the cooperative control signal (to be designed) that is responsible for achieving consensus between the vehicles, while *g*_*e, i*_(*t*) and *g*_ω, *i*_(*t*) represent secondary objectives, where *g*_*e, i*_(*t*) is an error correction term, responsible for delaying the evolution of the path variable in case of momentary vehicle failure, and *g*_ω, *i*_(*t*) is a rotation correction term, responsible for canceling the rotational motion induced on the path by the rotation of the target. These functions are to be properly defined in section 3.3.

## 3. Robust Cooperative Moving Path Following Control

### 3.1. Robust MPF Controller Design

In this section, we consider the kinematic controller proposed in Jain et al. (2018a) with a modification designed to ensure robustness against disturbances. For the *i*-th vehicle, the error dynamics is given by

$$\begin{array}{l} {\overset{∙}{e}}_{i}={\dot{R}}_{i}^{T}\left({\bar{p}}_{i}-p_{d,i}\right)+R_{i}^{T}\left({\bar{\dot{p}}}_{i}-{\dot{p}}_{d,i}\right). \end{array}$$

Using model (1) with control signal (2) and MPF error (5), the error dynamics can be rewritten as

(8) $$\begin{array}{l} {\overset{∙}{e}}_{i}=-S\left(\omega_{i}+d_{\omega ,i}\right)e_{i}+\Delta u_{i}+d_{i}-R_{i}^{T}v_{t}\\ \;-R_{i}^{T}R_{t}\left(\nabla p_{d,i}^{t}{\overset{∙}{\gamma }}_{i}+S\left(\omega_{t}\right)p_{d,i}^{t}\left(\gamma_{i}\right)\right) \end{array}$$

where Δ is a constant matrix that can take the forms

$$\Delta =\left[\begin{array}{cc} 1 & -\epsilon_{2}\\ 0 & \epsilon_{1} \end{array}\right]\text{ or }\Delta =\left[\begin{array}{cccc} 1 & 0 & \epsilon_{3} & -\epsilon_{2}\\ 0 & -\epsilon_{3} & 0 & \epsilon_{1}\\ 0 & \epsilon_{2} & -\epsilon_{1} & 0 \end{array}\right]$$

for the planar (*n* = 2) and 3D (*n* = 3) cases, respectively. Note that it is always possible to choose ϵ such that Δ is full rank. Vector $d_{i}\in {\text{R}}^{n}$ is the total external disturbance acting on the vehicle. In the planar case, it is given by

(9) $$\begin{array}{l} d_{i}=\left[\begin{array}{c} R_{i}^{T}{\;s}_{\epsilon } \end{array}\right]\left[\begin{array}{c} d_{v,i}\\ d_{\omega ,i} \end{array}\right]\\ s_{\epsilon }={\left[\begin{array}{c} -\epsilon_{2\;}\epsilon_{1} \end{array}\right]}^{T} \end{array}$$

Remark 3.1. *Notice that, by the triangle inequality, the total external disturbance* ***d***_*i*_ *is bounded by* ∥***d***_*i*_∥ ≤ ∥***d***_*v, i*_∥ + ∥***d***_ω, *i*_∥∥**ϵ**∥.

Assumption 3.1. *The total external disturbances* ***d***_*i*_ *are bounded vector quantities.*

Theorem 1 (**Robust MPF)**. *Consider an underactuated robotic vehicle described by (1) with control signal given by (2). Let the MPF error kinematics be described by (8), and consider that the pose of the* *i*-*th vehicle* $\left\{p_{i},R_{i}\right\}\in {\mathbb{R}}^{n}\times SO\left(n\right)$ and of the target frame $\left\{p_{t},R_{t}\right\}\in {\mathbb{R}}^{n}\times SO\left(n\right)$ *are known. Under Assumptions 2.1 and 2.2, the control law*

(10) $$\begin{array}{l} u_{i}=\Delta^{†}(-K_{p,i}e_{i}+R_{i}^{T}\left({\hat{v}}_{t}+R_{t}S\left({\hat{\omega }}_{t}\right)p_{d,i}^{t}\right)\\ \;+R_{i}^{T}R_{t}\nabla p_{d,i}^{t}{\overset{∙}{\gamma }}_{i}-w_{i}) \end{array}$$

(11) $$\begin{array}{l} w_{i}=\{\begin{array}{c} \rho_{i}\frac{e_{i}}{∥e_{i}∥},\;∥e_{i}∥\geq \epsilon_{w}\\ \rho_{i}\frac{e_{i}}{\epsilon_{w}},\;∥e_{i}∥<\epsilon_{w} \end{array} \end{array}$$

*ensures that all trajectories of the MPF error are globally uniformly ultimately bounded and converge to a ball around the origin* ***e***_*i*_ = *0 that can be made arbitrarily small. In (10), the matrix* Δ^†^ *is the Moore-Penrose pseudo-inverse of* Δ, $K_{p}\in {\text{R}}^{n\times n}$ *is a positive-definite gain matrix and* ${\hat{v}}_{t}\in {\mathbb{R}}^{n}$, ${\hat{\omega }}_{t}\in {\mathbb{R}}^{n\left(n-1\right)/2}$ *are estimates of the target velocities. In (11)*, ρ_*i*_ *is a scalar such that*

(12) $$\begin{array}{l} \rho_{i}\geq ∥d_{v,i}∥+∥d_{\omega ,i}∥∥\epsilon ∥+∥{\tilde{v}}_{t}∥+∥{\tilde{\omega }}_{t}\times p_{d,i}^{t}\left(\gamma_{i}\right)∥ \end{array}$$

*where* ${\tilde{v}}_{t}=v_{t}-{\hat{v}}_{t}$, ${\tilde{\omega }}_{t}=\omega_{t}-{\hat{\omega }}_{t}$ *are bounded estimation errors on the target velocities.*

*Proof*. Define the Lyapunov candidate $V\left(e_{i}\right)=\frac{1}{2}e_{i}^{T}e_{i}$. Using the error dynamics in (8), its time derivative along the system trajectories is

(13) $$\begin{array}{l} \overset{∙}{V}\left(e_{i}\right)=e_{i}^{T}\left(\Delta u_{i}+d_{i}-R_{i}^{T}v_{t}-R_{i}^{T}R_{t}\left(\nabla p_{d,i}^{t}{\overset{∙}{\gamma }}_{i}+S\left(\omega_{t}\right)p_{d,i}^{t}\right)\right) \end{array}$$

where we have used the fact that $e_{i}^{T}S\left(\omega_{i}+d_{\omega ,i}\right)e_{i}=0$, since *S*(**ω**_*i*_ + ***d***_ω, *i*_) is skew-symmetric. Substituting control law (10) in (13) yields

(14) $$\begin{array}{l} \overset{∙}{V}\left(e_{i}\right)=-e_{i}^{T}K_{p}e_{i}+e_{i}^{T}\left(D_{i}-w_{i}\right), \end{array}$$

where $D_{i}=d_{i}-R_{i}^{T}\left({\tilde{v}}_{t}+R_{t}S\left({\tilde{\omega }}_{t}\right)p_{d,i}^{t}\right)$. Since *K*_*p*_ > 0, the first term is negative definite and bounded by $-\lambda_{\text{min}}(K_{p})\;{\left\|e_{i}\right\|}^{2}$. Next, we consider the two cases of (11), when ∥***e***_*i*_∥ ≥ ϵ_*w*_ or ∥***e***_*i*_∥ < ϵ_*w*_.

- For ∥***e***_*i*_∥ ≥ ϵ_*w*_ in (14), we have
  $$\begin{array}{l} \dot{V}(e_{i})\leq -\lambda_{\text{min}}(K_{p}){\left\|e_{i}\right\|}^{2}+e_{i}^{T}D_{i}-\rho_{i}\frac{e_{i}^{T}e_{i}}{\left\|e_{i}\right\|}\\ \;\leq -\lambda_{\text{min}}(K_{p})\;{\left\|e_{i}\right\|}^{2}+\;\left\|e_{i}\right\|(\left\|D_{i}\right\|\text{​}-\text{​}\rho_{i}) \end{array}$$
  where the Cauchy-Schwarz inequality was employed on term $e_{i}^{T}D_{i}$. By Assumption 3.1, it is always possible to design ρ_*i*_ such that (12) is satisfied. Therefore, by Remark 3.1, choosing ρ_*i*_ ≥ ∥***D***_*i*_∥ renders the second term on the right-hand side negative definite, which estabilishes that the trajectory ***e***_*i*_(*t*) of the closed-loop system reaches the ball $B\left(\epsilon_{w}\right):=\left\{e_{i}\in {\mathbb{R}}^{n}:∥e_{i}∥\leq \epsilon_{w}\right\}$ in finite time.
- When the trajectories are inside $B\left(\epsilon_{w}\right)$, we have ∥***e***_*i*_∥ < ϵ_*w*_, and (14) gets
  $$\begin{array}{l} \dot{V}(e_{i})\leq -\lambda_{\text{min}}(K_{p}){\left\|e_{i}\right\|}^{2}+e_{i}^{T}D_{i}-\rho_{i}\frac{e_{i}^{T}e_{i}}{\epsilon_{w}}\\ \;\leq -(1-\theta )\lambda_{\text{min}}(K_{p}){\left\|e_{i}\right\|}^{2}\\ \;-\left(\theta \lambda_{\text{min}}(K_{p})+\frac{\rho_{i}}{\epsilon_{w}}\right){\left\|e_{i}\right\|}^{2}+\left\|e_{i}\right\|\left\|D_{i}\right\| \end{array}$$
  where 0 < θ < 1. Then, using the inequality above, one can write:
  $$\begin{array}{l} \dot{V}(e_{i})\leq -(1-\theta )\lambda_{\min}(K_{p}){\left\|e_{i}\right\|}^{2}<0\;\forall \left\|e_{i}\right\|\geq \mu_{i},\\ \;\mu_{i}=\frac{\left\|D_{i}\right\|\epsilon_{w}}{\lambda_{\min}(K_{p})\theta \epsilon_{w}+\rho_{i}} \end{array}$$
  Note that μ_*i*_ ≤ ϵ_*w*_ for all 0 < θ < 1, which means that the trajectory of the closed-loop system ***e***_*i*_(*t*) again reaches the ball $B\left(\mu_{i}\right)\subseteq B\left(\epsilon_{w}\right)$ in finite time.

This establishes that the trajectories are globally ultimately uniformly bounded, since $V=\frac{1}{2}\;{\left\|e_{i}\right\|}^{2}$ is radially unbounded (Khalil, 2002). Moreover, ***e***_*i*_(*t*) converges to the ball $B\left(\mu_{i}\right)\subseteq B\left(\epsilon_{w}\right)$, which can be made arbitrarily small when ϵ_*w*_ → 0.     □

### 3.2. Robust MPF Controller Design With Disturbance Observer

In the presence of large amplitude disturbances, it may be difficult to tune the parameters ρ_*i*_ and ϵ_*w*_ so as to satisfy (12). In these situations, an observer can be designed to provide an estimate of the disturbance. Furthermore, this estimate can be used in the control law to compensate the real disturbance directly.

Without loss of generality, consider the planar problem. Consider that the vehicle pose $\left\{p_{i},R_{i}\right\}\in {\mathbb{R}}^{2}\times SO\left(2\right)$ is known and that the vehicle orientation is parameterized by the planar angle ψ_*i*_ ∈ ℝ, such that $R_{i}=R_{i}\left(\psi_{i}\right)\in SO\left(2\right)$.

Then, the disturbance observer for the translational disturbance is defined as

(15) $$\begin{array}{l} \{\begin{array}{c} {\overset{∙}{\hat{p}}}_{i}=R_{i}v_{i}+{\hat{d}}_{v,i}+K_{1}{\tilde{p}}_{i}\\ {\overset{∙}{\hat{d}}}_{v,i}=K_{2}{\tilde{p}}_{i}, \end{array} \end{array}$$

where the estimation errors are defined as ${\tilde{p}}_{i}=p_{i}-{\hat{p}}_{i}$ and ${\tilde{d}}_{v,i}=d_{v,i}-{\hat{d}}_{v,i}$, and the positions ***p***_*i*_, i∈I are accurately measured. For positive-definite matrices $K_{1},K_{2}\in {\mathbb{R}}^{2\times 2}$, the dynamics of the estimation errors ${\tilde{p}}_{i}$, ${\tilde{d}}_{i}$ can be proven to be Input-to-State Stable (ISS) with respect to the first time-derivative of ***d***_*v, i*_ (Aguiar and Pascoal, 2002).

Similarly, observers for the rotational disturbances *d*_ω, *i*_ ∈ ℝ can be designed as:

(16) $$\begin{array}{l} \{\begin{array}{c} {\overset{∙}{\hat{\psi }}}_{i}=\omega_{i}+{\hat{d}}_{\omega ,i}+k_{\omega_{1}}{\tilde{\psi }}_{i}\\ {\overset{∙}{\hat{d}}}_{\omega ,i}=k_{\omega_{2}}{\tilde{\psi }}_{i}, \end{array} \end{array}$$

where the estimation errors are defined as ${\tilde{\psi }}_{i}=\psi_{i}-{\hat{\psi }}_{i}$ and ${\tilde{d}}_{\omega ,i}=d_{\omega ,i}-{\hat{d}}_{\omega ,i}$, and the planar angles ψ_*i*_ are measured. Again, for positive scalars *k*_ω_1__, *k*_ω_2__ ∈ ℝ_>0_, the dynamics of the estimation errors ${\tilde{\psi }}_{i}$, ${\tilde{d}}_{\omega ,i}$ can be proven to be ISS with respect to the first time-derivative of *d*_ω, *i*_ (Aguiar and Pascoal, 2002).

Theorem 2 (**Robust MPF with Disturbance Observer)**. *Consider an underactuated robotic vehicle described by (1) and control signal given by (2). Let the MPF error kinematics be described by (8), and consider that the pose of the vehicle* $\left\{p_{i},R_{i}\right\}\in {\mathbb{R}}^{n}\times SO\left(n\right)$ *and of the target frame* $\left\{p_{t},R_{t}\right\}\in {\mathbb{R}}^{n}\times SO\left(n\right)$ *are known. Under Assumptions 2.1 and 3.1, the control law*

(17) $$\begin{array}{l} u_{i}=\Delta^{†}(-K_{p,i}e_{i}+R_{i}^{T}\left({\hat{v}}_{t}+R_{t}S\left({\hat{\omega }}_{t}\right)p_{d,i}^{t}\right)\\ \;+R_{i}^{T}R_{t}\nabla p_{d,i}^{t}{\overset{∙}{\gamma }}_{i}-w_{i}-{\hat{d}}_{i}) \end{array}$$

*ensures that all trajectories of the MPF error are globally uniformly ultimately bounded and converge to a ball around the origin* ***e***_*i*_ = *0 that can be made arbitrarily small. In (17), matrix* Δ^†^ *is the Moore-Penrose pseudo-inverse of* Δ, $K_{p}\in {\mathbb{R}}^{n\times n}$ *is a positive-definite gain matrix*, ${\hat{v}}_{t}\in {\mathbb{R}}^{n}$ *and* ${\hat{\omega }}_{t}\in {\mathbb{R}}^{n\left(n-1\right)/2}$ *are estimates of the target velocities and* ${\hat{d}}_{i}$ *is the total estimated external disturbance, which is a function of the states of the disturbance observers*

(18) $$\begin{array}{l} {\hat{d}}_{i}=\left[\begin{array}{c} R_{i}^{T}s_{\epsilon } \end{array}\right]\left[\begin{array}{c} {\hat{d}}_{v,i}\\ {\hat{d}}_{\omega ,i} \end{array}\right] \end{array}$$

The term ***w***_*i*_ is defined by (11), with scalars ρ_*i*_ satisfying

(19) $$\begin{array}{l} \rho_{i}\geq ∥{\tilde{d}}_{v,i}∥+|{\tilde{d}}_{\omega ,i}|∥\epsilon ∥+∥{\tilde{v}}_{t}∥+∥{\tilde{\omega }}_{t}\times p_{d,i}^{t}\left(\gamma_{i}\right)∥ \end{array}$$

*Proof*. The proof is very similar to Theorem 1, and can be performed by proposing the same Lyapunov candidate $V=\frac{1}{2}e_{i}^{T}e_{i}$. Differentiating it in time and applying the error dynamics (8) with control law (10) yields

(20) $$\begin{array}{l} \overset{∙}{V}\left(e_{i}\right)=-e_{i}^{T}K_{p}e_{i}+e_{i}^{T}\left({\tilde{D}}_{i}-w_{i}\right) \end{array}$$

where ${\tilde{D}}_{i}={\tilde{d}}_{i}-R_{i}^{T}\left({\tilde{v}}_{t}+R_{t}S\left({\tilde{\omega }}_{t}\right)p_{d,i}^{t}\right)$ and ${\tilde{d}}_{i}$ is the total estimation error defined by ${\tilde{d}}_{i}=d_{i}-{\hat{d}}_{i}$.

Note that (20) is similar to (14), but with disturbance ${\tilde{D}}_{i}$ instead of ***D***_*i*_. Therefore, using the same arguments for the proof of Theorem 1 with Assumption 2.2 and condition (19), one can conclude that the trajectories of the MPF error are globally uniformly ultimately bounded and ***e***_*i*_(*t*) converges to the ball $B\left({\bar{\mu }}_{i}\right)\subseteq B\left(\epsilon_{w}\right)$, which can be made arbitrarily small when ϵ_*w*_ → 0.     □

Remark 3.2. *Comparing conditions (12) and (19) for the choice of* ρ_*i*_, *in (19) the gain* ρ_*i*_ *must overcome only the norm of the disturbance estimation errors instead of the norm of the disturbance. Therefore, if the disturbance observer is properly designed, this method can reduce the necessary amount of control effort when compared to the previous method.*

Remark 3.3. *Both proposed control laws (10, 17) employ estimates of the target velocities. Since the velocity estimation errors* ${\tilde{v}}_{t}$ *and* ${\tilde{\omega }}_{t}$ *appear as additional disturbances in* ***D***_*i*_ *and* ${\tilde{D}}_{i}$, *they can be properly compensated by the proposed controllers as long as* ρ_*i*_ *satisfies (12) or (19). In this case, the velocity estimation errors are implicitly assumed to be bounded. Furthermore, notice that in the case where no velocity estimators are employed* (${\hat{v}}_{t}=0$ *and* ${\hat{\omega }}_{t}=0$), *the velocity estimation errors are simply* ${\tilde{v}}_{t}=v_{t}$ *and* ${\tilde{\omega }}_{t}=\omega_{t}$, *which are also bounded. These observations imply that velocity estimators are not necessarily required for the implementation of the proposed control laws. However, large velocity estimation errors would increase the lower bounds for the design of* ρ_*i*_, *increasing the amount of control effort, which could lead to loss of performance.*

### 3.3. Cooperative Moving Path Following

This section provides a proper design for function ϑ_*i*_ in (7). First, the design of the error correction term *g*_*e, i*_(*t*) and of the rotation correction term *g*_ω, *i*_(*t*) are discussed, and finally we make use of the results from Olfati-Saber et al. (2007) to design a cooperative control law *v*_*r, i*_(*t*).

#### 3.3.1. Error Correction Term

The term *g*_*e, i*_(*t*) is a bounded error correction term that acts as an external input to the path dynamics, enabling faster convergence of the robotic vehicle to the moving path. It can be designed to delay or to stop the motion of the virtual point if the vehicle is too far away from the path. This can be done by defining the gradient with respect to the path variable of the MPF error norm squared:

(21) $$\begin{array}{l} \eta_{e,i}=\nabla \left(\frac{1}{2}e_{i}^{T}e_{i}\right)=-e_{i}^{T}R_{i}^{T}R_{t}\nabla p_{d,i}^{t}\left(\gamma_{i}\right) \end{array}$$

and then choosing a gradient descent law *g*_*e, i*_ = − *k*_*e, i*_*sat*(η_*e, i*_) with *k*_*e, i*_ > 0. The saturation function guarantees the boundedness for the correction term. Its effect is to effectively delay the evolution of the virtual point along the path by explicitly avoiding the evolution of γ_*i*_ if the MPF error norm is too large.

#### 3.3.2. Path Rotation Correction Term

The term *g*_ω, *i*_(*t*) is designed to delay the evolution of the virtual point ***p***_*d, i*_ in a such a way that minimizes the effect of the target rotational motion, which is evident from the term $S\left(\omega_{t}\right)p_{d,i}^{t}\left(\gamma_{i}\right)$ in (4). This effect is important since, for large target angular velocities **ω**_*t*_, the virtual point could move faster than the *i*-th vehicle could reach. Therefore, substituting (7) into the error dynamics (8), we seek to design a scalar *g*_ω, *i*_ such that

(22) $$\begin{array}{l} g_{\omega ,i}\left(t\right)=\underset{g_{i}\in \mathbb{R}}{arg min}∥\nabla p_{d,i}^{t}\left(\gamma_{i}\right)g_{i}+S\left(\omega_{t}\right)p_{d,i}^{t}\left(\gamma_{i}\right)∥. \end{array}$$

If the target angular velocity is known, the minimum can be achieved by the least squares solution

(23) $$\begin{array}{l} g_{\omega ,i}(\omega_{t},\gamma_{i})=-\frac{\nabla^{T}p_{d,i}^{t}S(\omega_{t})p_{d,i}^{t}}{{\left\|\nabla p_{d,i}^{t}\right\|}^{2}}, \end{array}$$

with minimum given by

$$\underset{g_{i}\in \mathbb{R}}{\min}\;\left\|\nabla p_{d,i}^{t}(\gamma_{i})g_{i}+S(\omega_{t})p_{d,i}^{t}(\gamma_{i})\right\|=\frac{{\left(\nabla p_{d,i}^{t}\right)}^{T}p_{d,i}^{t}}{{\left\|\nabla p_{d,i}^{t}\right\|}^{2}}S(\omega_{t})\nabla p_{d,i}^{t}.$$

Remark 3.4. *Note that the minimum is identically null regardless the rotational motion of the target only if and only if: (i) the path is perpendicular to its gradient everywhere, i.e.*, ${\left(\nabla p_{d,i}^{t}\right)}^{T}p_{d,i}^{t}=0\forall \gamma_{i}$, i∈I *or (ii) the angular velocity of the target is collinear to the path gradient everywhere, i.e.*, $\omega_{t}=c\nabla p_{d,i}^{t}=0\forall \gamma_{i}$, i∈I *for some constant* *c* ∈ ℝ. *Clearly, condition (ii) never holds in the planar case* (*n* = *2)*.

Assumption 3.2. *The path gradients are non-vanishing everywhere, i.e.*, $\nabla p_{d,i}^{t}\left(\gamma_{i}\right)\neq 0,\forall \gamma_{i},i\in I$.

From (23) and Assumption 3.2, the error correction term is bounded by.

$$\begin{array}{l} |g_{\omega ,i}\left(t\right)|\leq \frac{\max_{\gamma_{i}}∥p_{d,i}^{t}\left(\gamma_{i}\right)∥}{\min_{\gamma_{i}}∥\nabla p_{d,i}^{t}\left(\gamma_{i}\right)∥}∥\omega_{t}∥. \end{array}$$

#### 3.3.3. Cooperative Controller

Consider the distributed consensus law (Aguiar, 2017):

(24) $$\begin{array}{l} v_{r,i}=-k_{c,i}\underset{j\in N_{i}}{\sum }\left(\gamma_{i}-{\hat{\gamma }}_{j}^{i}\right),\;\forall i\in I \end{array}$$

where *k*_*c, i*_ > 0 are consensus gains and ${\hat{\gamma }}_{j}^{i}$ are estimates of the path variables of the neighbor vehicles ($\gamma_{j},j\in N_{i}$) running inside the *i*-th vehicle computer. Assuming that the frequency of communication is low, its reasonable to assume that ${\hat{\gamma }}_{j}^{i}\neq \gamma_{j},\forall t>0$. Therefore, one can write ${\hat{\gamma }}_{j}^{i}=\gamma_{j}-{\tilde{\gamma }}_{j}^{i}$, where ${\tilde{\gamma }}_{j}^{i}$ is a path variable estimation error.

Assumption 3.3. *Given a fixed, undirected communication topology between the vehicles, the* *i*-*th vehicle updates its path variable* γ_*i*_ *to its* $j\in N_{i}$ *neighbors in a fixed frequency. Additionally, assume that no data package is lost during communication. Consequently, the path variable estimation errors* ${\tilde{\gamma }}_{j}^{i},\forall i,j\in I$ *are always bounded.*

Define the vectors $\gamma ={\left[\begin{array}{c} \gamma_{1}\gamma_{2}\cdots \gamma_{N} \end{array}\right]}^{T}$, $g_{e}={\left[\begin{array}{c} g_{e,1}g_{e,2}\cdots g_{e,N} \end{array}\right]}^{T}$, $g_{\omega }={\left[\begin{array}{c} g_{\omega ,1}g_{\omega ,2}\cdots g_{\omega ,N} \end{array}\right]}^{T}$and $1_{N}={\left[\begin{array}{c} 1\;1\cdots 1 \end{array}\right]}^{T}\in {\mathbb{R}}^{N}$. Using (24) in (7) and stacking the dynamic equations, one can write

(25) $$\begin{array}{l} \overset{∙}{\gamma }=v_{d}1_{N}-K_{c}L\gamma -K_{c}\tilde{\gamma }+g_{e}+g_{\omega }, \end{array}$$

where *K*_*c*_ = *diag*(*k*_*c*, 1_, *k*_*c*, 2_, …, *k*_*c, N*_) is a positive definite matrix of consensus gains, *L* = *D* − *A* ∈ ℝ^*N* × *N*^ is the *Laplacian* of the network connection graph, defined by $D=diag\left(\left|N_{1}|,|N_{2}|,\ldots ,|N_{N}\right|\right)$ and the *adjacency matrix* *A* = [*a*_*ij*_], with *a*_*ij*_ = 1 if $j\in N_{i}$ and *a*_*ij*_ = 0 otherwise. Vector $\tilde{\gamma }$ is defined as ${\left[\tilde{\gamma }\right]}_{i}=\underset{j\in N_{i}}{\sum }{\tilde{\gamma }}_{j}^{i}$, i.e., its *i*-th element is the sum of all path variable estimation errors for the *i*-th vehicle.

Theorem 3 (**Cooperative Controller)**. *Consider a fleet of* *N* *underactuated robotic vehicles with dynamics described by (1) and control signal given by (2). Then, control laws (10) or (17) with robustness term (11) guarantee that the origin of the MPF error* ***e***_*i*_ ≡ *0 is stable under the same conditions and assumptions of Theorems 1 and 2, respectively.*

*Furthermore, under Assumption 3.3, the cooperative control law given by (24) ensures that* $|\gamma_{i}-\gamma_{j}|,\forall i,j\in I$ *are Input-to-State Stable (ISS)*^1^ *with respect to the path variable estimation errors* ${\left[\tilde{\gamma }\right]}_{i}$, *error correction terms* *g*_*e, i*_ *and rotation correction terms* *g*_ω, *i*_, ∀i ∈ I.

*Proof*. The first part of the Theorem was already proved in Theorems 1 and 2. The part related to the cooperative control follows the same core ideas from (Jain et al., 2018a). First, define the *disagreement vector* (Olfati-Saber et al., 2007) as **δ**: = **γ** − α**1**_*N*_, with $\alpha =\left(1/N\right)1_{N}^{T}\gamma$.

Note that the consensus condition $|\gamma_{i}-\gamma_{j}|=0,\forall i,j\in I$ is achieved if and only if **δ** = **0**. Additionally, the following two properties hold: (i) *L***γ** = *L***δ** and (ii) $1_{N}^{T}\delta =0$.

Next, define the ISS Lyapunov function candidate

$$V_{cc}\left(\delta \right)=\delta^{T}L\delta \geq 0$$

Taking its time-derivative and using (25), yields

(26) $$\begin{array}{l} {\overset{∙}{V}}_{cc}=-z^{T}K_{c}z-z^{T}K_{c}\tilde{\gamma }+z^{T}g_{e}+z^{T}g_{\omega } \end{array}$$

with **z** = *L***δ**, where we used the properties (i) and (ii) introduced before. Using the Cauchy-Schwartz inequality, yields

(27) $$\begin{array}{l} {\dot{V}}_{cc}\leq -\lambda_{\text{min}}(K_{c}){\left\|z\right\|}^{2}+\lambda_{\text{max}}(K_{c})\left\|z\right\|\left\|\tilde{\gamma }\right\|+\left\|z\right\|\left\|g_{e}\right\|+\left\|z\right\|\left\|g_{\omega }\right\| \end{array}$$

Applying Young's inequality to the last three terms in (27), we have

$$\begin{array}{l} {\dot{V}}_{cc}\leq -\left(\lambda_{\text{min}}(K_{c})-\frac{\lambda_{\text{max}}(K_{c})}{2c}-\frac{1}{2c}-\frac{1}{2c}\right){\left\|z\right\|}^{2}\\ \;+\frac{c\lambda_{\text{max}}(K_{c})}{2}{\left\|\tilde{\gamma }\right\|}^{2}+\frac{c}{2}{\left\|g_{e}\right\|}^{2}+\frac{c}{2}{\left\|g_{\omega }\right\|}^{2} \end{array}$$

with a scalar *c* ∈ ℝ_>0_. Choosing any $c>\frac{\lambda_{\text{max}}\left(K_{c}\right)}{2\lambda_{\text{min}}\left(K_{c}\right)}+\lambda_{\text{min}}^{-1}\left(K_{c}\right)>0$ leaves the first term of the right-hand side strictly negative, which by Assumption 2.4 and by the boundedness of *g*_*e, i*_, *g*_ω, *i*_ establishes that the disagreement vector **δ** is ISS with respect to the bounded disturbances ${\left[\tilde{\gamma }\right]}_{i}$, *g*_*e, i*_ and *g*_ω, *i*_, for all i∈I.     □

## 4. Experimental Results

### 4.1. Experimental Setup

**Figure 2:**
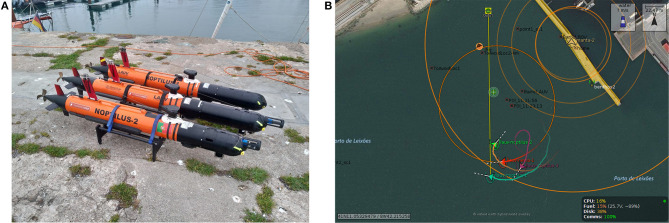
**(A)** Autonomous underwater vehicles used on the experiments. **(B)** The Neptus console.

The experiments were performed on Porto de Leixões (Porto, Portugal) using three Light Autonomous Underwater Vehicles (LAUVs) from the Underwater Systems and Technology Laboratory (LSTS) at the Faculty of Engineering of the University of Porto (FEUP) (Figure 2A). LAUVs are lightweight, portable vehicles that can be easily launched, operated and recovered with a minimal operational setup.

The vehicles operate under the DUNE/Neptus environments, which are part of a software toolchain (Pinto et al., 2013) developed and maintained by LSTS. DUNE is the on-board software running on the vehicles, comprising all the software needed for communications, navigation, control, maneuvering, plan execution and supervision of multiple types of robotic vehicles. The control algorithms were implemented on C++, using the available DUNE libraries. Neptus is a software used for command, control and monitoring, comprising many typical functions needed for a typical mission, such as planning, execution and post-mission analysis (Figure 2B).

A target vehicle was *simulated* and continuously sends its position and orientation (computed from GPS/IMU measurements using an extended Kalman filter (Braga et al., 2012) to the three follower vehicles through static UDP connections with a maximum frequency of 1*Hz*. The control algorithm for the target vehicle is a vector field method (Nelson et al., 2007) that is responsible to steer the vehicle along a circumference with radius equal to 60*m* in the clockwise direction at 0.5*m*/*s*. The desired moving paths for the follower AUVs are planar circumference centered at the target vehicle with phase difference of 2π/3 between them:

(28) $$\begin{array}{l} p_{d,i}^{t}\left(\gamma_{i}\right)=R\left[\begin{array}{c} \cos\left(\gamma_{i}/R+\phi_{i}\right)\\ \sin\left(\gamma_{i}/R+\phi_{i}\right) \end{array}\right], \end{array}$$

where *R* = 25*m*, ϕ_1_ = 0 *rad*, ϕ_2_ = 2π/3 *rad* and ϕ_3_ = − 2π/3 *rad*. Each vehicle sends its path variable to the neighbor vehicles with a frequency of 1*Hz* to maintain coordination, according to the consensus law (24) and Assumption 2.4. The consensus gains are $k_{c,i}=0.1,\forall i\in I$.

For the construction of the MPF errors ***e***_*i*_, the value **ϵ** = [1 0]^*T*^ was used. The controller gain matrices and error correction gains were chosen as *K*_*p, i*_ = *diag*(0.2, 0.2) and $k_{e,i}=2,\forall i\in I$. The reference for the path variable velocity is *v*_*d*_ = 1*m*/*s*.

Remark 4.1. *We point out the fact that this particular kind of vehicles cannot generate reliable negative forward velocities due to its propeller design. Given the fact that control laws (10), (17) can generate negative forward velocities if the virtual point is behind the line-of-sight of the vehicle, a substitute controller was designed to override the original controller in case this happens.*

*Therefore, while the forward velocity generated by (10) or (17) is negative* (*v*_*f, i*_ < *0), the applied control signal will be*

$$\begin{array}{l} u_{i}=\left[\begin{array}{c} v_{C}\\ -sgn\left({\left[e_{i}\right]}_{y}-\epsilon_{2}\right)\omega_{C} \end{array}\right] \end{array}$$

*instead, until (10), (17) generate a positive* *v*_*f, i*_ *again. Constants* *v*_*C*_, ω_*C*_ ∈ ℝ *are strictly positive. That means that the vehicle performs a “turning” maneuver with constant velocities until the virtual point is once again inside its line-of-sight. The direction of the turn is clockwise if the virtual point is to the right of the vehicle and counterclockwise if the virtual point is to the left of the vehicle. This strategy allows arbitrary initial configurations of the vehicles with respect to the initial position of the virtual point, and also allows the vehicles to recover from practical dead lock situations where their line-of-sight is kept facing away from the virtual point, which could happen, for example, in case of communication losses. In this case*, *v*_*C*_ = *1.7m*/*s* *and* ω_*C*_ = *1rad*/*s*, *approximately the upper saturation limits for the actuators.*

### 4.2. Experimental Results

#### 4.2.1. CMPF With Velocity Compensation

**Figure 3:**
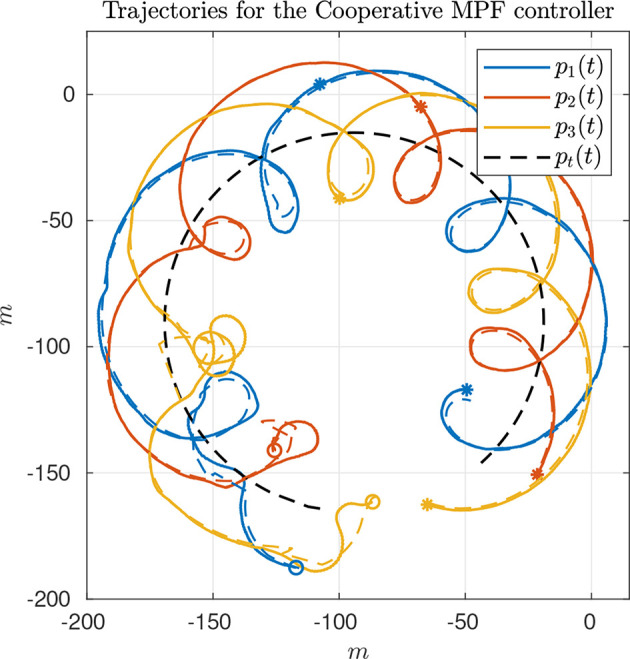
Vehicle trajectories for the CMPF controller with velocity compensation.

**Figure 4:**
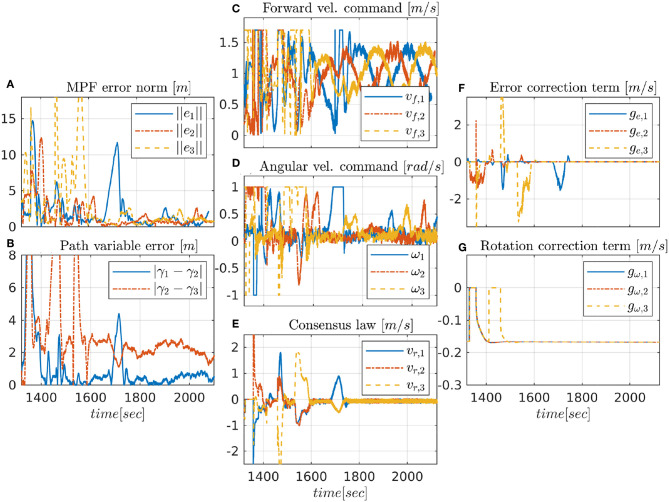
Experimental results for the CMPF controller with velocity compensation.

The first experiment shows the results of the CMPF controller with velocity compensation, ρ_*i*_ = 0 and no disturbance compensation (${\hat{d}}_{v,i}=0$ and ${\hat{d}}_{\omega ,i}=0$). Figure 3 shows the trajectories of the vehicles. The trajectory of the target is represented as the dashed black circle, in the clockwise direction. The small colored circles represent the beginning of the trajectory, while the colored asterisks represent its end. Noticeably, the three vehicles try to follow their respective paths (shown in dashed lines) around the rotating target, while maintaining their phase difference. Figure 4 shows the obtained results. The initial position of the vehicles was distant from the network router (located closer to the northeast part of Figure 3), which affected the wireless communications for a while. However, the initially large path variable errors rapidly decrease and remain bounded to less than 4 m (Figure 4B). Because of the communication losses and possibly the presence of ocean currents, the secondary controller described in Remark 3.5 had to recover some vehicles during the transient, resulting in some of the turning maneuvers we see in the beginning of the trajectories (Figure 3). After that transient, the norm of the MPF errors converge to a small region of less than 3 m while the control signal remains inside its linear region (Figures 4C,D). Note how the consensus law acts precisely when the path variable errors are high (Figure 4E), how the error correction terms acts when the MPF error norm is high (to prevent the evolution of the path variables), and how the rotation correction terms is fixed to a small value (≈ 0.18*m*/*s*) during the whole experiment. This is due to the fact that the target moves with constant angular velocity and the paths are circles to all three vehicles (see 23).

#### 4.2.2. Robust CMPF With Sliding Mode Term

**Figure 5:**
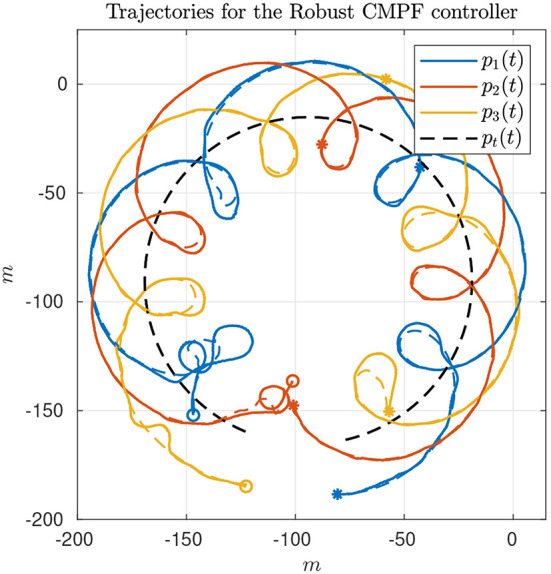
Vehicle trajectories for the robust CMPF controller.

**Figure 6:**
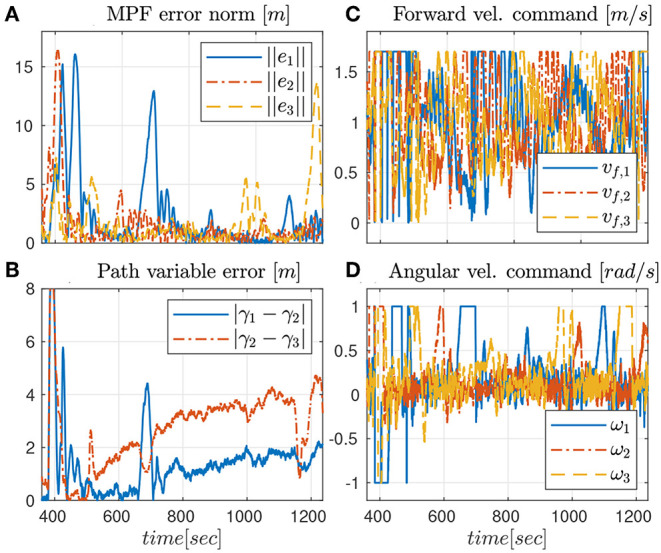
Results for the robust CMPF controller.

The second experiment shows the results of the robust CMPF controller with velocity compensation and Sliding Mode term, with MPF control law given by (10) with ρ_*i*_ = 0.2 for the three vehicles and ϵ_*w*_ = 0.5*m*. The consensus law for cooperation among the vehicles is given by (24), as before. Figure 5 shows the vehicle trajectories around the target, starting and ending in the southwest and southeast corners, respectively. Once more, due to communication losses and the presence of ocean currents in the southwest location, the secondary controller described in Remark 3.5 was activated for some of vehicles during the transient. However, the proposed controller was able to stabilize the error faster than the nominal controller. Besides, from Figure 6A, it is possible to notice the practical sliding mode phenomena around the origin ***e***_*i*_ ≡ 0. That means that the controller is able to achieve better performance than the previous one, given that ϵ_*w*_ can be designed to be arbitrarily small. However, from (11), small values of ϵ_*w*_ can result in higher gains for ***w***_*i*_, which can potentially saturate the control inputs. In fact, sometimes the control saturation limits are reached after the transient, as shown in Figures 6C,D, and practical sliding mode is momentarily lost. The reason is the limited velocity range allowed by the actuators, combined with our particular value choice for ϵ_*w*_, and moments of occasional increase in the target velocity. Even so, performance is slightly better than in the previous case, and the amount of control chattering is acceptable.

The consensus law, error correction signals and rotation correction signals are omitted, but are similar to those observed in Figure 4.

#### 4.2.3. Robust CMPF With Sliding Mode Term and Disturbance Compensation

The third and last experiment shows the results of the robust CMPF controller with velocity compensation, Sliding Mode term and direct disturbance compensation using a linear observer. The control law is given by (17) with ρ_*i*_ = 0.2 and ϵ_*w*_ = 0.5*m*, as before. Again, the consensus law for cooperation among the vehicles is given by (24).

**Figure 7:**
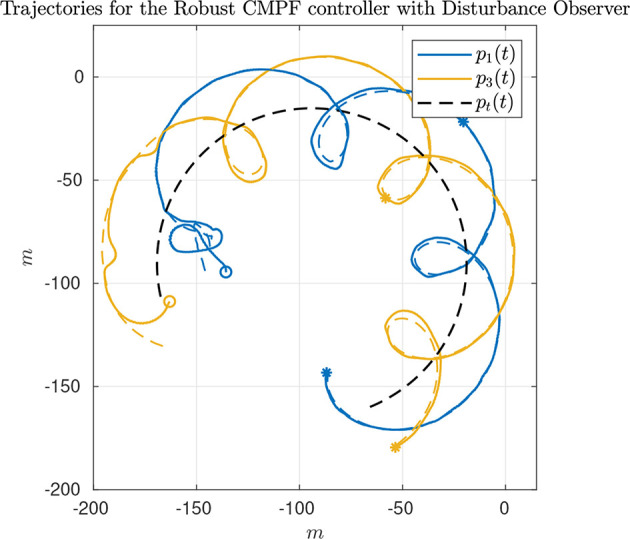
Vehicle trajectories for the robust CMPF controller with disturbance observer.

**Figure 8:**
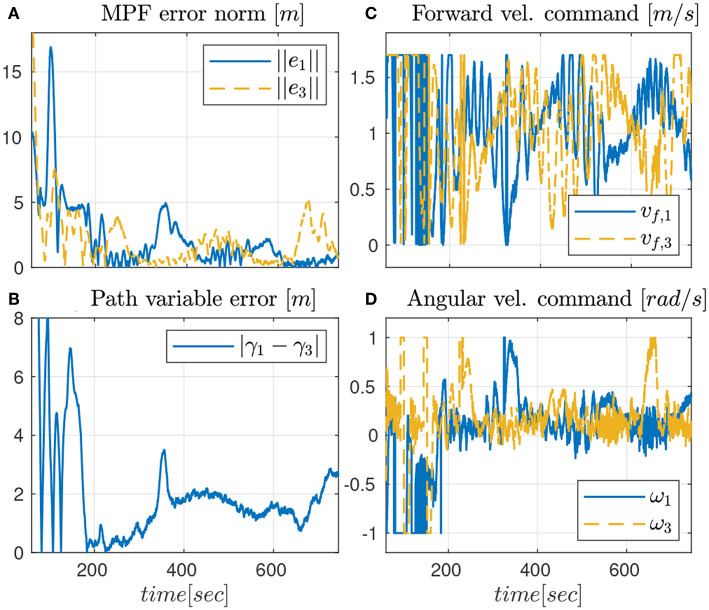
Results for the robust CMPF controller with disturbance observer.

**Figure 9:**
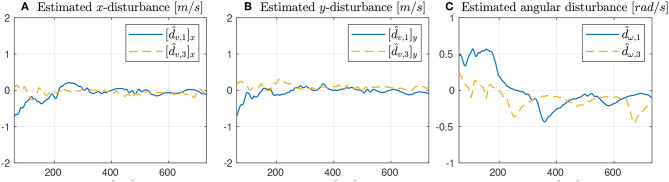
Results obtained with the disturbance estimator.

As seen from Figure 7, only the vehicles Noptilus 1 and 3 were used on this experiment, since the battery on Noptilus 2 was depleted. However, the results obtained by Noptilus 1 and 3 can still be compared to the previous results obtained for the same two vehicles. The chosen paths are the same circles defined in (28), but this time with ϕ_1_ = 0 and ϕ_3_ = π*rad*. This modification was used to guarantee that the two vehicles stay as far as possible from each another. Once again, in Figure 8A, notice the practical sliding mode phenomena around the origin ***e***_*i*_ ≡ 0, except during the instants where the control inputs are saturated (Figures 8C,D). However, in this case, the control chattering is significantly smaller than the one observed in Figures 6C,D, under the same experimental conditions. We explain this fact by the presence of the disturbance estimator. Since part of the disturbance is compensated, the sliding mode term can spend less effort compensating the remaining total disturbance, a result compatible with the theoretical insight of Remark 3.2. The path variable errors remain bounded by 4m, as shown in Figure 8B. The estimated disturbances are shown in Figure 9. The linear velocity disturbances remained bounded by < 0.3*ms* after the transient, while the angular velocity disturbances showed higher variation, but remained bounded to < 0.5*rads* after the transient.

## 5. Conclusions

This work addressed the robust cooperative MPF problem for marine vehicles. We demonstrated that the origin of the MPF errors associated to the vehicles are stable with the two proposed robust CMPF control schemes in the presence of bounded disturbances acting on the vehicles. Furthermore, it was theoretically demonstrated that the cooperative control scheme is ISS with respect to the path variable estimation errors and to two other bounded, auxiliary input variables, named error correction term and rotation correction terms. The proposed robust controllers (10, 17) guarantee that the MPF error is globally uniformly bounded to a small neighborhood of the origin while maintaining acceptable control chattering. The narrow linear region of the actuators imposes limits on how small ϵ_*w*_ can be designed in practice. Lastly, we conclude that control law (17) actually improved the control chattering in practice, corroborating the theoretical insight of Remark 3.2.

Some of the future works are: (i) to investigate how to extend the proposed controllers to the case of unknown bounds for the disturbances (ii) to take the existence of actuator saturation limits in the control design and (iii) to incorporate obstacle avoidance techniques into the cooperative MPF approach to prevent vehicle collision during the cooperation tasks.

## Data Availability Statement

The datasets generated for this study are available on request to the corresponding author.

## Author Contributions

MR has written the manuscript, implemented the algorithms in C++ code, and performed the experiments using the LSTS vehicles. He also proposed the sliding mode based scheme for adding robustness to the moving path following controllers. RJ has proposed the cooperative control scheme using a consensus law, and contributed significantly to the stability proof of the cooperative controller. He also helped by suggesting important changes on the code and with the organization of the manuscript. AA contributed with the proposition of the disturbance compensation method for improving the performance of the first controller (Theorem 2), and also strongly contributed to the stability proofs and overall organization of the paper. JS made the experiments possible by setting up the mission at Porto de Leixões and has contributed by suggesting some changes on the code.

### Conflict of Interest

The authors declare that the research was conducted in the absence of any commercial or financial relationships that could be construed as a potential conflict of interest.

## Footnotes

^1^Khalil (2002) A nonlinear system $\overset{∙}{\delta }=f\left(t,\delta ,\epsilon \right)$ is said to be Input-to-State Stable (ISS) if there exist a class KL function β and a class K function γ such that for any initial state **δ**(*t*_0_) and any bounded input **ϵ**(*t*), the solution **δ**(*t*) exists for all *t* ≥ *t*_0_ and satisfies

$$∥\delta \left(t\right)∥\leq \beta \left(∥\delta \left(t_{0}\right)∥,t-t_{0}\right)+\gamma \left(\underset{t_{0}\leq \tau \leq t}{\sup}\;∥\epsilon \left(\tau \right)∥\right)$$
